# COVID-19 serological survey using micro blood sampling

**DOI:** 10.1038/s41598-021-88850-z

**Published:** 2021-05-04

**Authors:** Melissa M. Matthews, Tae Gyun Kim, Satoshi Shibata, Noriko Shibata, Christian Butcher, Jaekyung Hyun, Keon Young Kim, Theodore Robb, Siang Sheng Jheng, Masashi Narita, Tomoari Mori, Mary Collins, Matthias Wolf

**Affiliations:** 1 Molecular Cryo-Electron Microscopy Unit, Okinawa Institute of Science and Technology Graduate University (OIST), Onna-son, Okinawa Japan; 2 Fluid Mechanics Unit, OIST, Onna-son, Okinawa Japan; 3 IT Division, Infrastructure Section, OIST, Onna-son, Okinawa Japan; 4 Division of Infectious Diseases, Okinawa Chubu Hospital, Okinawa City, Okinawa Japan; 5 Research Support Division, Occupational Health and Safety, OIST, Onna-son, Okinawa Japan; 6 Research Support Division, Office of the Provost, OIST, Onna-son, Okinawa Japan; 7 Institute of Biological Chemistry, Academia Sinica, Taipei, Taiwan

**Keywords:** Cryoelectron microscopy, Viral infection, Epidemiology, Occupational health, Viral infection

## Abstract

During August 2020, we carried out a serological survey among students and employees at the Okinawa Institute of Science and Technology Graduate University (OIST), Japan, testing for the presence of antibodies against SARS-CoV-2, the causative agent of COVID-19. We used a FDA-authorized 2-step ELISA protocol in combination with at-home self-collection of blood samples using a custom low-cost finger prick-based capillary blood collection kit. Although our survey did not find any COVID-19 seropositive individuals among the OIST cohort, it reliably detected all positive control samples obtained from a local hospital and excluded all negatives controls. We found that high serum antibody titers can persist for more than 9 months post infection. Among our controls, we found strong cross-reactivity of antibodies in samples from a serum pool from two MERS patients in the anti-SARS-CoV-2-S ELISA. Here we show that a centralized ELISA in combination with patient-based capillary blood collection using as little as one drop of blood can reliably assess the seroprevalence among communities. Anonymous sample tracking and an integrated website created a stream-lined procedure. Major parts of the workflow were automated on a liquid handler, demonstrating scalability. We anticipate this concept to serve as a prototype for reliable serological testing among larger populations.

## Introduction

At the beginning of 2020, COVID-19 emerged from Hubei province in southern China and quickly spread across the globe. Due to its proximity and direct flight connections between the epicenter in Wuhan and its capital Tokyo, the Japanese Nation was among the first to experience cases of the disease outside China, documenting its first case on January 15, 2020. In early April, as the tourism season in Okinawa began to ramp up, so did the new cases of COVID-19 in Okinawa. A first outbreak of COVID-19 cases in mid-March (total number 132 cases) was effectively quenched by 5 weeks of lock-down measures to no additional cases within the next two months. From early June on, however, a second much stronger wave of infections has culminated in more than 8445 accumulated cases on the island (as of March 15, 2021). The present study was conducted in August 2020.

Serological surveys which detect the presence of antibodies against SARS-CoV-2 antigens can provide important information to the government for issuing health care guidelines. At the beginning of April 2020, we obtained plasmids for coronavirus surface antigens from the Krammer Lab at the Icahn School of Medicine (NY, NY, USA). We established protein expression and purification in a mammalian cell line and set up an ELISA following the 2-step assay developed by the same group^1^. Their assay has received emergency use authorization by the U.S. Food and Drug Administration (FDA)^2^. We secured PCR-confirmed human sera from COVID-19 positive patients at the local hospital as well as negative controls from serum collected before December 2019. Once the assay itself was validated, we set up partially automated sample handling on a robotic liquid handler, established a website with a barcoding system for anonymous sample tracking, and conducted a serological survey of staff and students at our institution.

In emergency situations such as during COVID-19, the health care system is under stress; it cannot be expected that trained clinical personnel are available to draw patient blood by venous puncture. Furthermore, non-essential human traffic in hospitals and other health care institutions should ideally be limited to protect vital health care workers from risk of exposure to potential carriers of the virus. To overcome this limitation, we distributed easy-to-use, self-administered micro blood sampling kits to participants. The kit uses a single-use safety lancet to collect a few drops of capillary blood from the participant’s finger. We show that antibody titers obtained by micro blood sampling are equivalent to serum antibody titers from blood drawn by conventional venous puncture. This low-cost, easily deployable self-sampling method in combination with a highly sensitive and specific ELISA in a centralized testing lab provides a scalable solution that can enable serological surveys of larger populations.

## Results

### Protein preparation and validation

**Figure 1:**
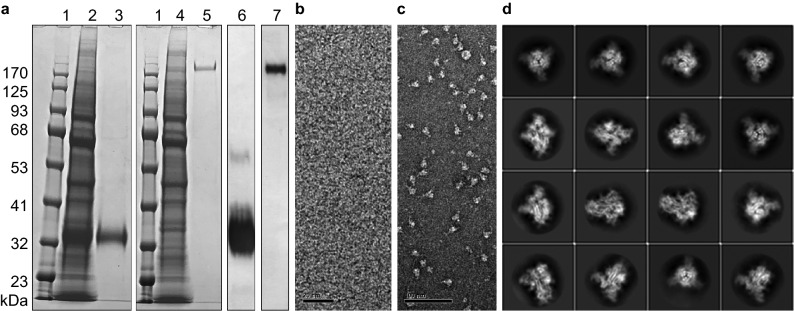
(**a**) SDS-PAGE of expressed and purified RBD and spike proteins. Lane: 1, molecular weight marker; 2, cell lysate of expressed RBD; 3, purified RBD; 4, cell lysate of expressed SARS-CoV-2 S; 5, purified spike; 6 and 7, Western blot of purified RBD and spike, respectively. (**b**,**c**) Electron micrographs of negatively stained purified RBD and spike protein, respectively. Scale bars 20 nm 100 nm, respectively. (**d**) 2D class averages from boxed aligned single-particle cryo-EM images of the trimeric spike protein, showing secondary structure elements indicative of proper folding. Box size 28 nm. The class averages were created with RELION 3.1^23^. Original images are available as supplementary data files.

SARS-CoV-2 trimeric spike and its receptor-binding domain (RBD) were expressed in mammalian cells and purified by chromatography. The quality was verified by Western blot and electron microscopy (Fig. 1a–c). We performed single particle cryo-EM and 2D-image classification. This confirmed that the trimeric spike protein was properly folded and assembled (Fig. 1d).

### Validation of micro blood sampling method

Serum samples are typically separated from intravenously collected blood. To confirm that the finger-prick method neither causes unforeseen complications nor affects assay sensitivity, blood samples from two confirmed SARS-CoV-2 PCR-positive individuals were taken both intravenously and by finger-prick on the same day. Although these samples were collected at least 92 days post exposure, they retained a high antibody titer comparable to positive controls, which were collected 10–30 days post onset of symptoms.

**Figure 2:**
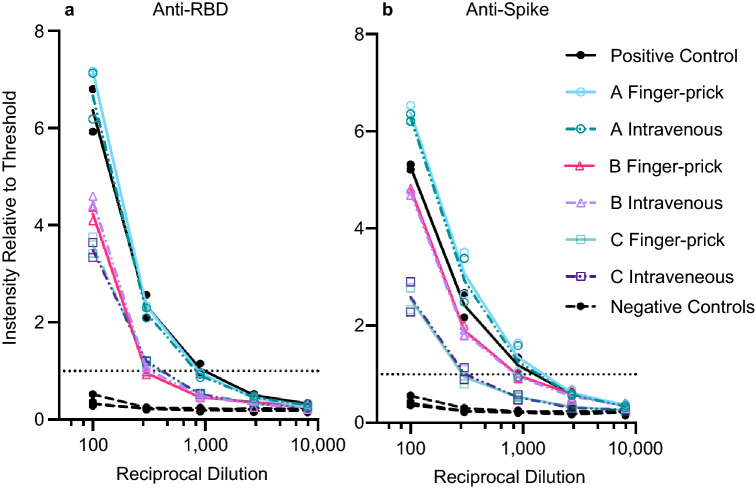
Serum antibody titers from intravenously collected blood and capillary blood obtained by finger prick are equivalent. Individuals A, B and C provided blood samples both intravenously and also by micro-blood collection at least 70 days post exposure. Each sample was tested in duplicate on the same ELISA plate, either coated with SARS-CoV-2 spike RBD (left) or with SARS-CoV-2 trimeric spike protein (right). Each replicate is plotted. Line connects average values. Positive control was pooled serum collected from two SARS-CoV-2 PCR-positive individuals 10–30 days after onset of symptoms. Negative controls were collected from individuals prior to November 2019.

The MiniCollect capillary blood collection tube contains a prefilled volume of a polymer gel with a lithium heparin coagulant, which induces blood clotting and can separate the blood clot from serum by centrifugation. Separated serum from four samples were tested in a dilution series in duplicate on both an RBD-coated ELISA plate and a trimeric spike-coated ELISA plate according to the “step 2” ELISA protocol^1^ (see Methods and modified plate layout in Supplementary Figure S1c). For both individuals, titers of intravenous and capillary blood were similar on both the RBD-coated and the spike-coated plate (Fig. 2).

### Serological survey

Overall, 675 sample tubes were collected and processed. Among all the samples received, zero samples showed signal above threshold in both the RBD screening plate and the spike confirmatory plate.

Samples were typically collected, serum separated, and heat-inactivated at the end of each day. Nonetheless, most blood samples can be stored at room temperature in the serum separation tube for several days, in some cases with larger blood volume for up to 1 week. Longer than 1 week is not recommended because the blood begins to dry up. Antibody titers after serum separation have been reported as stable for up to 6 weeks when stored at 4 °C^3^. Some participants had difficulty collecting their blood by themselves. In such cases, we encouraged participants to visit the nurses in our institute’s Health Center for assistance.

**Figure 3:**
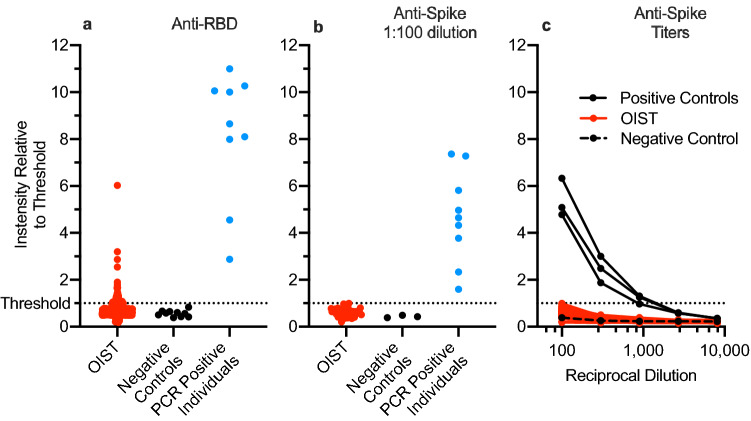
Summary of OIST Serological Survey. (**a**) In assay step 1 (anti-RBD ELISA), 63 OIST samples were above the threshold (3 standard deviations above the average negative control). (**b**) All positives from step 1 were tested in step 2 (anti-spike ELISA). No OIST samples were above the threshold (4 times the average background). (**c**) Step-2 titers were measured at dilutions 1:100, 1:300, 1:900, 1:2700, and 1:8100. Positive controls showed much stronger signal than the OIST samples even at high dilution. The dotted line indicates the respective threshold level. All negative controls are samples taken from patients prior to November 2019. Sera from PCR positive individuals were collected at least 10–30 days post onset of symptoms.

The results for RBD ELISA step 1 are summarized in Fig. 3a. Intensities were scaled with respect to the threshold for each plate. Results were compared with multiple negative controls and a collection of serum samples from SARS-CoV-2 PCR positive individuals. A subset of 63 OIST samples had serum antibody reactivity above the established threshold, and only a few were at the same level as the PCR-positive individuals. Titers from all PCR-positive individuals were above the threshold. Samples from all PCR-positive individuals were taken by capillary blood collection at least one month post onset of symptoms.

Results for the final ELISA step 2 are shown in Fig. 3b,c. Of the 63 OIST samples that were positive in RBD step 1, all had serum antibody reactivity below threshold for all 5 dilutions when tested against the SARS-CoV-2 trimeric spike protein in step 2, indicating a 9.9% false positive rate in the highly sensitive step 1. Antibody titers (Fig. 3c) of serum samples from all PCR positive individuals were above the threshold for at least two dilutions. Results were classified as “positive” (above threshold), “negative” (below threshold), or “undetermined”. All RBD-positive OIST samples were below threshold in the anti-spike ELISA (Fig. 3b,c). The most common cause of an undetermined result was the failure to provide sufficient serum. This was the case for 40 of the 675 samples that we received. If the result was undetermined, the participant was encouraged to pick up another kit and try again.

From the SurveyMonkey platform, 206 completed surveys were received, or 31% of all samples collected. The collective results showed that 17% of the survey takers believed that they had experienced some COVID-19 symptoms in the past 6 months and 31% had travelled outside of Okinawa in the past 6 months. Of the 63 participants that showed signal above threshold against RBD, only 10 responded to the optional survey. 3 of those 10 reported that they had experienced some COVID-19 symptoms in the past 6 months. The age distribution was as follows: 20–40 years old, 60%; 40–60 years, 36%; greater than 60 years, 4%. The gender distribution was roughly equal (Supplementary Table ST1).

### Assay specificity

**Figure 4:**
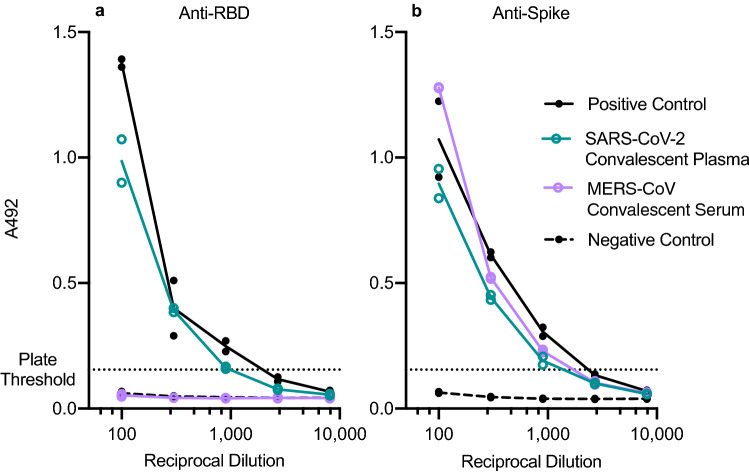
Cross-reactivity of MERS convalescent serum with SARS-CoV-2 spike protein. Line connects average ELISA antibody titer values. Each sample was tested in duplicate on the same plate. Positive control is pooled serum from two CoV-2 PCR positive individuals 10–30 days after onset of symptoms. Negative control is from a commercial serum pool.

The specificity of the Mount Sinai Hospital Clinical Laboratory COVID-19 ELISA Antibody Test has been reported as 100% for 74 negative control samples^4^ and the developers demonstrated no cross-reactivity against the common coronavirus strain NL63^5^. Of note, as part of the protocol establishing process, we used a SARS-CoV-2 convalescent plasma (NIBSC code 20/130) and human MERS-convalescent serum to confirm the specificity of the assay in both steps. As expected, SARS-CoV-2 convalescent plasma showed strong reactivity in both plates. MERS-convalescent serum, on the other hand, was negative in anti-RBD cross-reactivity, but positive in anti-spike cross-reactivity (Fig. 4). To understand the basis of the MERS-convalescent serum cross-reactivity, we performed an amino acid sequence alignment of SARS-CoV-2 spike and MERS-CoV spike protein. Sequence alignment demonstrated that the SARS-CoV-2 RBD shares 18.7% identity with MERS-CoV RBD, but alignment of the full-length spike sequence demonstrated higher 32.3% sequence identity between SARS-CoV-2, and MERS-CoV (Supplementary Figure S3).

### Longevity of antibody titers

**Figure 5:**
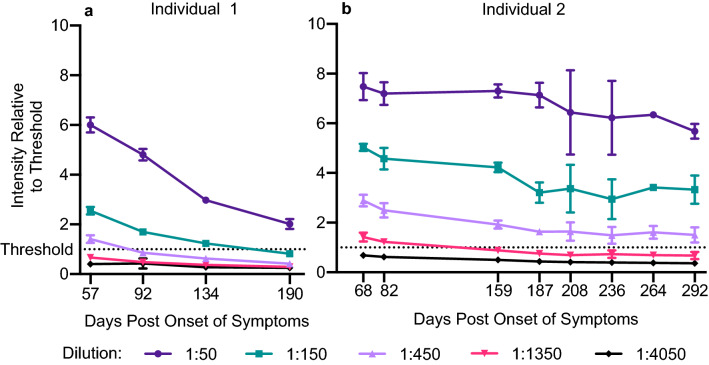
Resiliency of anti-spike antibody titer over time. Serum samples from two recovered COVID-19 patients were collected at 57, 92, 134, and 190 days post onset of symptoms (Individual 1, left) or at 68, 82, 159, 187, 208, 236, 264, and 292 days post onset of symptoms (Individual 2, right). Anti-spike antibody titers were measured by ELISA at 50, 150, 450, 1350, and 4050 times dilution in quadruplicate (Individual 1, Days 57 and 92; Individual 2, Days 68, 82, 159, and 187) or in duplicate (Individual 1, Days 134 and 190; Individual 2, Days 208, 236, 264, and 292). The average and standard deviation for each dilution of each sample is plotted, revealing detectable antibody titers at 1:450 dilution for more than 9 months post onset of symptoms for Individual 2, with the day of symptoms onset being day 0.

We obtained multiple samples from two PCR-confirmed SARS-CoV2 positive individuals over an extended period of time and analyzed their serum antibody titers against the spike protein by ELISA. Antibody titer was measured by anti-spike ELISA in a series of dilutions (Fig. 5). Anti-spike antibodies titer remained above threshold for more than 9 months post onset of symptoms.

## Discussion

### Micro blood sampling is a good alternative to venous blood collection

Micro blood sampling by finger prick at home avoids unnecessary risk and effort by healthcare workers and allows parallel collection of large numbers of samples within a short period of time. The combination of patient-driven sample self-collection with a standardized highly specific and selective ELISA in a centralized lab improves the reliability of results while keeping costs low. Serum can be separated from whole blood up to 1 week after collection. Provided that temperatures are kept close to ambient temperature, this would allow for shipping of samples from collection site to the testing site by mail. The main bottleneck is serum transfer from collection tube to ELISA plate. This step may be accelerated with a sophisticated integrated liquid handling system. We showed that antibody titers from capillary blood serum are equivalent to titers measured from venous blood (Fig. 2). The Infectious Diseases Society of America Guidelines on the Diagnosis of COVID-19 currently make no recommendations for or against the use of capillary blood in serological assays due to a knowledge gap on the subject^6^. We hope that the success of our study begins to fill this knowledge gap, paving the way for further proof-of-concept studies using capillary blood. Our survey revealed that micro blood sampling of capillary blood is a practical, cost-efficient method using as little as one drop of blood (approximately 30 µL) per sample. Such a small sample volume would allow testing of newborns using neonatal heel prick.

### Anonymization and web-based reporting

The web-based platform served as an efficient means to conduct this study anonymously while providing instructions and communicating results to participants. Anonymization was achieved by randomly barcoded samples without the need to enter personal data. From the 1154 staff and students at OIST (as of May 2020), 675 samples were received, of which only 41 samples (6.1%) could not be tested due to lack of serum or improper sample collection. The success of our practical web-based method indicates that this method may be used for larger epidemiological studies.

### Significance of OIST results

The serological survey of students and employees carried out at OIST on Okinawa, Japan, in August 2020 revealed no seroconversion among this small population. The test has reliably identified all previously confirmed PCR-positive individuals and controls, with reactivities as high as 7 times threshold. Our results assert strong confidence in this 2-step assay, which has received FDA-emergency approval^2^. Although we have not validated our assay against another commercial ELISA-based kit, we have used two rapid diagnostic tests (Kurabo SARS-CoV-2, lateral flow rapid diagnostic test against IgG and IgM) on 18 randomly selected survey participants, including one of the RBD positive individuals. All of these rapid test results were negative, whereas the IgG rapid test did correctly detect our positive controls.

A key issue that had to be addressed throughout the establishment of the assay was how to calculate the cutoff thresholds for each plate. Our step 1 plate was designed so as to have internal negative controls which were used to calculate the threshold for each plate separately. This method worked well to sensitively select a subset of samples to be tested more specifically in step 2. A few samples showed anti-RBD reactivity at levels similar to that of serum samples from individuals with known history of CoV-2 infection, but were found to be negative against the CoV-2 spike protein. Given that reactivity against RBD at this level has been seen among SARS-CoV-2 naïve control samples previously^7^, we have confidence these samples were correctly determined to be negative for CoV-2 seropositivity.

Okinawa has, thus far, enjoyed moderately low numbers of COVID-19 cases among its population. Other recent serological surveys in Japan, have found seroprevalence as low as 0.43% (among 44,066 employees and business partners of the company Softbank and healthcare workers across Japan)^8^, 0.1% (1971 citizens of Tokyo), 0.03% (3009 citizens in Miyagi Prefecture) and 0.17% (2970 citizens of Osaka prefecture)^9^. At such low seroprevalence, high assay specificity is critical to achieving a high predictive value^10,11^. Although accurate estimation of the actual seroprevalence among OIST staff and students (including the 45% of employees that did not participate in the survey) is not possible, assuming a seroprevalence between 0.03% (Tokyo) and 0.43% (Softbank), with a known assay specificity of close to 100% and a sensitivity of 92.5% for the Mt. Sinai Antibody Test used here^4^, we estimate the negative predictivity value of our assay to be greater than 99.9%.

### Longevity of antibody titers

Analysis of serum antibody titers against SARS-CoV2 spike protein taken at multiple time points from two PCR-positive individuals post onset of symptoms (or post verified exposure) revealed only moderate decrease over time, with robust antibody titers above threshold persisting for more than 9 months (Fig. 5). In combination with recent reports of high efficacy of vaccines currently in phase-3 trials, our results add to the growing amount of evidence that spike-based vaccines may be able to elicit longer-lasting immunity.

### Cross-reactivity of MERS convalescent serum with SARS-CoV-2 spike protein

SARS-CoV, SARS-CoV-2 and other human coronaviruses such as NL63 use aceE2 as receptor^12,13^ mediated by the class-1 fusion protein S before entry through the plasma membrane, or via a clathrin-dependent endosomal pathway^14^. MERS-CoV utilizes a similar mechanism but uses the dipeptidyl peptidase 4 receptor (dpp4) instead^15^. Dpp4 does not share sequence and structural similarity to previously identified human coronavirus receptors such as ACE2 or APN^12,13,16^. Convalescent plasma samples from SARS-CoV-infected patients have moderate cross-reactivity with SARS-CoV-2 spike protein, but no cross-neutralization^17^. The assay used here has been shown to have no cross-reactivity with the seasonal human coronavirus NL63^5^. Interestingly, when we tested human convalescent serum from MERS patients with our ELISA, we found explicit cross-reactivity between MERS serum and SARS-CoV-2 spike with an antibody titer similar to that of SARS-CoV-2 plasma. Our sequence alignment indicated only 18.7% sequence identity between SARS-CoV-2 RBD and MERS-CoV RBD. On the other hand, we found 32.3% identity between SARS-CoV-2 and MERS-CoV spike (S) (Supplementary Figure S3), indicating higher sequence conservation in the other domains of the spike protein, thus providing a possible explanation for the observed cross-reactivity. For a given SARS-CoV-2 convalescent serum sample with an anti-spike titer of > 1:1350, the probability of viral neutralization at the FDA-recommended level for convalescent plasma used for COVID-19 treatment (viral neutralization titer ≥ 1:160), has been found to be ≥ 80%^18^. Our data shows strong MERS cross-reactivity with SARS-CoV-2 spike at titers close to 1:1350. Although most effective neutralizing antibodies against coronaviruses target the RBD, neutralizing antibodies against SARS-CoV-2 S1-N-terminal domain^19^ and SARS-CoV S2 domain^20^ have also been identified. Our data suggests that MERS convalescent serum may also contain such neutralizing antibodies against SARS-CoV-2.

### Translational utility of antibody tests using capillary blood

The ongoing pandemic has put an immense burden on hospitals and medical personnel. Micro-blood sampling using capillary blood allows for sample collection by individuals at home, without putting health care workers at risk. In the U.S.A., a large antibody survey using a similar principle has been carried out while distributing and collecting sample kits by mail^21^. The processing of such samples in a central lab using a highly sensitive and specific ELISA-based assay may provide a more accurate picture of the immunological status within population groups than less reliable rapid diagnostic tests. Using our own finger-prick based test kit, we have tested medical professionals, veterinarians, firefighters and members of other professional organizations. Further applications are antibody surveys of newborns using neonatal heel prick, and the quantification of antibody titers within the population post vaccination to validate vaccine action in individuals.

## Methods

### Protein expression and purification

Plasmids for mammalian expression of SARS-CoV-2 S (spike) protein receptor-binding domain (RBD, residues 319–541) and of stabilized His-tagged SARS-CoV-2 S including T4 foldon trimerization tag were generously provided by Florian Krammer (Icahn School of Medicine, NY, USA)^5^. These included SΔcs (furin cleavage site deletion RRAR86 to A) and SΔcspp (cleavage site deletion and stabilizing mutations K986P and V987P). Proteins were expressed in Expi293F cells and purified as described. Yield was 15 mg/L for RBD, and 4 mg/L for trimeric SARS-CoV-2 S. MonoRab Anti-His tag antibody (Nr. 25B6E11, GenScript, USA) was used for Western blotting.

### Electron microscopy

Protein particles were stained with 1% uranyl acetate on a carbon-coated copper grid and visualized with a Talos L120C transmission electron microscope (TEM) (Thermo Fisher Scientific, USA) (TFS) operating at 120 kV.

For cryo-EM, 3 µL purified trimeric spike sample solution at 2–3 mg/mL was applied to UltrAuFoil R1.2/1.3 grids pre-treated with a Solarus plasma system (Model 950, Gatan, USA) for 60 s at 25 °C in a 23% H_2_, 77% O_2_ gas mix. Grids were blotted and vitrified in a Vitrobot Mark IV (TFS) using a liquid ethane-propane mixture at liquid nitrogen temperature. Digital micrographs were captured using a Titan Krios cryo-TEM (TFS) operating at 300 kV on a Falcon-3EC camera (TFS) in counting mode, at a pixel size of 1.08 Å at the specimen level. Motion correction of the movies was done with MOTIONCOR2^22^. Contrast transfer function (CTF) estimation, particle picking and 2D classification were performed with RELION 3.1^23^. A total of 1,119,504 particles were picked from 1479 micrographs, of which 297,144 particles were retained after 2D classification.

### Sample collection and heat inactivation

Using a collection kit distributed by the researchers (Supplementary Figure S1a), participants collected 1–5 drops (approximately 30–150 µL) of capillary blood by self-finger prick with a safety lancet in a Greiner Bio-One MiniCollect serum separation tube (see Supplementary Video). Sera were separated by centrifugation at 5000×*g* for 5 min at 4 °C under biosafety level 2 (BSL2) conditions and heat-inactivated in a water bath at 56 °C for 1 h. Heat-inactivated samples were stored at 4 °C until use for ELISA. After scanning tube barcodes for each plate into a CSV file with a hand-held scanner, serum was transferred into barcoded 96-well plates by manual pipetting.

### ELISA and automation

We followed the 2-step ELISA protocol developed by the Krammer lab^1^. ELISA plate formats were modified as depicted in Supplementary Figure S1c. Modified plate designs included two dilution series of positive control, which provided an internal standard. Additional negative controls were included to provide a cutoff that was consistently within the suggested range of 0.15–0.2 AU (Absorbance Units) at 492 nm.

We implemented a partially automated workflow on a Beckman Biomek FX^P^ (Beckman Coulter, Indianapolis, IN, USA) liquid handling robot for two steps of the ELISA protocol, namely, for (i) dilution and transfer of serum samples for RBD screening plates and for (ii) plate developing and reading of all plates. Automated dilution and transfer of serum samples utilized a 96-well plate containing 5X diluted serum in PBS, termed the D1 (dilution 1) plate as a source plate. Sample from the D1 plate was diluted with a PBS-T milk solution in a second 96-well plate, termed the D2 (dilution 2) plate, and finally transferred to the destination ELISA plate according to the published protocol^1^. After dilution, D1 plates were immediately wrapped in parafilm and stored at 4 °C. If a given sample tested positive against RBD antigen in step 1, a fresh 5 × diluted sample was prepared using solution remaining in the serum separation tubes. If the volume of the remaining serum was insufficient, the 5 × diluted sample from the D1 plate was used for the confirmatory step 2.

For automated plate development and reading, SigmaFast OPD development solution (Sigma Aldrich) was used according to manufacturer instructions. The liquid handler was programmed to quench the reaction with 3 M HCl exactly 10 min after addition of OPD and immediately transfer each plate to a DTX800 multimode plate reader (Beckman Coulter, Indianapolis, IN, USA) for reading at 492 nm wavelength. All other steps, including washing on an AquaMax 4000 plate washer (Molecular Devices LLC, San Jose, CA, USA), were carried out manually.

### Controls and standards

Three positive controls in the form of intravenous blood sera from confirmed SARS-CoV-2 PCR-positive patients (collected 10–30 days post onset of symptoms) were obtained from Naha Municipal Hospital, Naha, Japan. After titer analysis of all three samples (data not shown), the two samples with the strongest titers were pooled and used as the positive control for all assays. Positive controls (collected at least 90 days after onset of symptoms) for validation of the capillary blood collection method were obtained from Okinawa Chubu Hospital, Uruma City, Japan. Negative controls taken from patients prior to November 2019 were obtained from Naha Municipal Hospital from intravenous blood, and from a commercial serum pool (Human Serum from human male AB plasma, Sigma Aldrich H4522-100ML, Batch #SLCD1948, serum was pooled prior to August 2019). Human MERS-convalescent serum and SARS-CoV-2 convalescent plasma (NIBSC code 20/130) were obtained from the National Institute of Biological Standards and Control, UK.

### Calculation of thresholds

The threshold for each step 1 plate was defined as the average of the negative controls plus 3 standard deviations of the negative controls^1^. The average and median threshold for all step 1 plates was 0.161 AU and 0.166 AU, respectively. Initially, the threshold for the step 2 plate was calculated in the same manner as step 1. However, the threshold calculated with only three negative controls was below the recommended range (0.15–0.2 AU at 492 nm). Therefore, another threshold, calculated as 4-times the average blank, which has been demonstrated to be valid for identifying anti-SARS-CoV-2 antibody-positive samples was used instead^24^. Average and median threshold for all step 2 plates were 0.196 AU and 0.1868 AU, respectively. Estimated predictivity values were calculated as described^25^.

### Website-based platform for test application and anonymous results reporting

Test results and detailed information about the experiment were disseminated using a custom web application deployed through Microsoft Azure Cloud (Supplementary Figure S2). Optional information about age, gender, prior symptoms, and travel history could be entered anonymously through the SurveyMonkey (https://www.surveymonkey.com) platform and linked to the sample ID which was passed as a hidden field via the participant’s web browser. Detailed information on ID methodology can be found in the Supplemental Information.

### Sequence alignments

The full-length sequences of SARS-CoV, MERS-CoV S protein (UniProt ID: P59594 and K9N5Q8, respectively) were aligned pairwise using ClustalW2^26^, against the SΔcs sequence^5^ (Supplementary Figure S3).

### Figure preparation and digital processing

Images of protein gels and Western blot in Fig. 1a were acquired by smartphone camera, cropped and adjusted for intensity level in Photoshop 2020, and assembled with Illustrator CC (Adobe Inc., USA). Original unaltered images (Fig. 1) are available as supplementary files. Graphs in Figs. 2, 3, 4 and 5 were plotted with GraphPad Prism v8.4.3 (GraphPad Software, USA). The 2D class averages (Fig. 1d) were created with RELION 3.1^23^. Our web application and database were hosted and deployed through Microsoft Azure cloud computing services. The instructional video was recorded on Apple iPhone 11 pro, edited with iMovie for macOS (Apple Inc., USA) and compressed for web viewing with HandBrake 0.10.2 software (The HandBrake Team, GNU GPL, https://handbrake.fr/).

### Ethics, consent, and permissions

The experiments were conducted according to a proposal approved by the OIST Human Subjects Research Review Committee (Protocol Title: “Survey of antibody retention rate for OIST staff and students against SARS-CoV-2”; application reference number: HSR-2020-026). All methods, including obtaining informed consent, were conducted in accordance with the Declaration of Helsinki and other relevant guidelines including the Ethical Guidelines for Medical and Health Research Involving Human Subjects set forth by the Japanese government. Consent to publish the accumulated anonymized data has been obtained from all participants of the study upon enrollment. The protocols for serum from PCR positive individuals, validation of micro blood sampling, testing of resiliency of anti-spike antibody titer over time, and other positive control samples have been approved by the Institutional Review Board (IRB) of Okinawa Chubu Hospital with the confirmation numbers 2020-23 and 2020-74-2, as well as Okinawa Nanbu Medical Center confirmation number R2-104. This protocol is furthermore included in the application with reference number HSR-2020-16-2 approved by the OIST Human Subjects Research Review Committee (Protocol Title: “Establishment of new serological diagnostic test by ELISA to grasp the post-infection population with novel coronavirus in Okinawa”).

## Supplementary Information

Supplementary Video.

Supplementary Information.

## Data Availability

Original uncropped unscaled images for Fig. 1a–d are available as supplementary files. Requests for further information or raw data should be directed to the corresponding author.
