# Supplementary material for: COVID-19 serological survey using micro blood sampling
Source: Sci Rep. 2021 May 4;11:9475. doi: 10.1038/s41598-021-88850-z (PMC8097019; doi:10.1038/s41598-021-88850-z)
Supplement: Supplementary file 2 — Supplementary Information. [file 41598_2021_88850_MOESM2_ESM.pdf]

Supplementary Information to "COVID-19 serological survey using micro blood sampling"

Melissa M. Matthews, Tae Gyun Kim, Satoshi Shibata, Noriko Shibata, Christian Butcher, Jaekyung Hyun, Keon Young Kim, Theodore Robb, Siang Sheng Jheng, Masashi Narita, Tomoari Mori, Mary Collins & Matthias Wolf

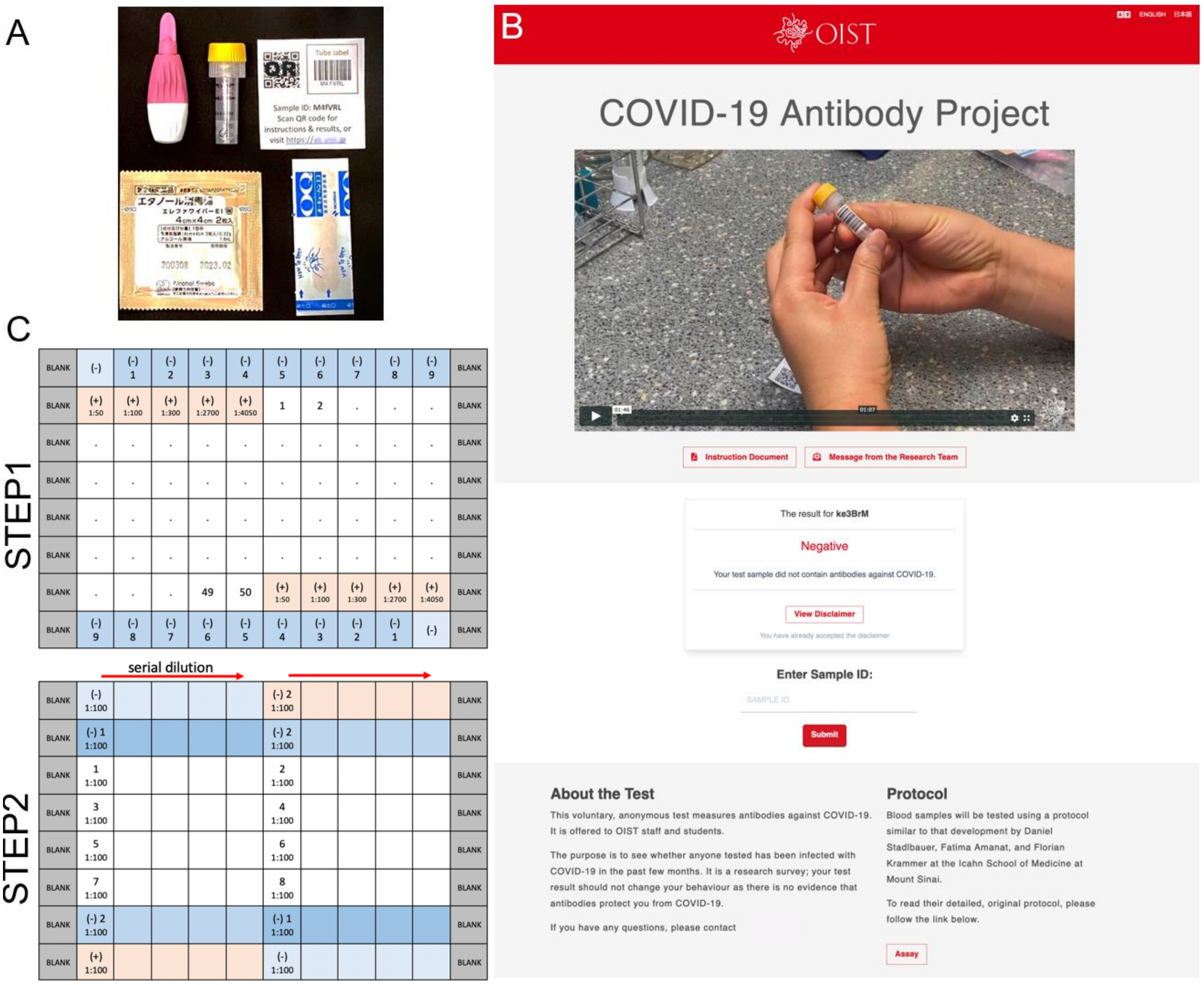

Supplementary Figure S1: Antibody Survey Sample Collection Kit and Plate Design. a. Each micro blood sampling kit included BD Microtainer contact-activated lancet (Becton Dickison, USA), 0.8 ml volume blood collection tube containing a coagulant and a separator (Greiner Bio-One MiniCollect TUBE 0.5/0.8 CAT Serum Sep Clot Activator gold cap), packaged alcohol wipes, adhesive bandage and sticker with an identification barcode (Code 128) and QR code, in 8 x 12 cm sealable plastic bag. The QR code encoded a unique URL containing the sample ID. Participants adhered the QR code to the collection tube before returning their sample. **b.** Front page of OIST Antibody Test website. Instructional video is available as supplementary file. **c.** Plate designs used in the ELISA. Plates are modified versions of those described previously (*1*).

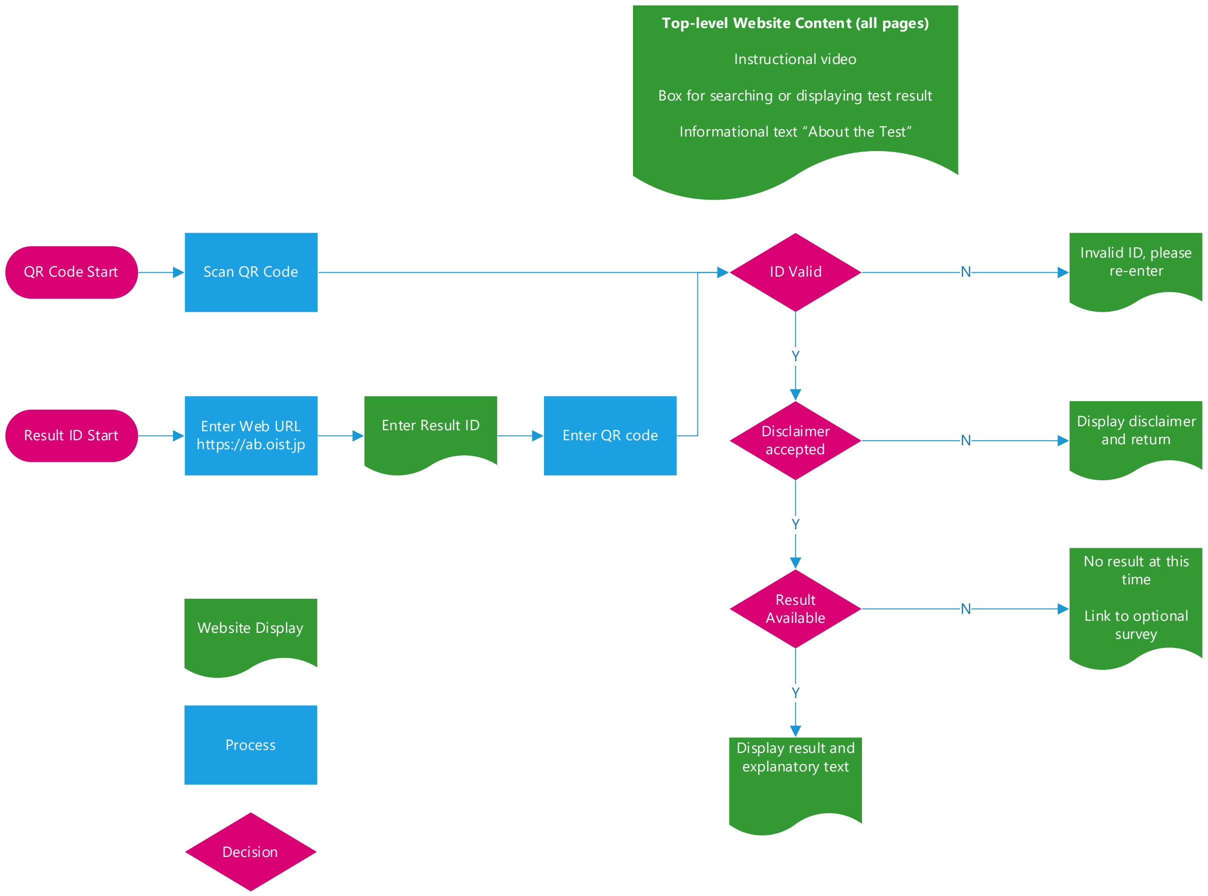

Supplementary Figure S2: Basic user flow followed by participants to view test results and answer optional survey. The diagram was created with Microsoft Visio Professional. Participants could check their test results anonymously either by scanning the QR code with a smartphone or by entering the 6-digit sample ID matching the linear tube barcode. Upon entering a valid sample ID, participants were prompted to accept a disclaimer before viewing results. After accepting the disclaimer, participants were able to retrieve the status of their sample, including guidance on interpreting test results.

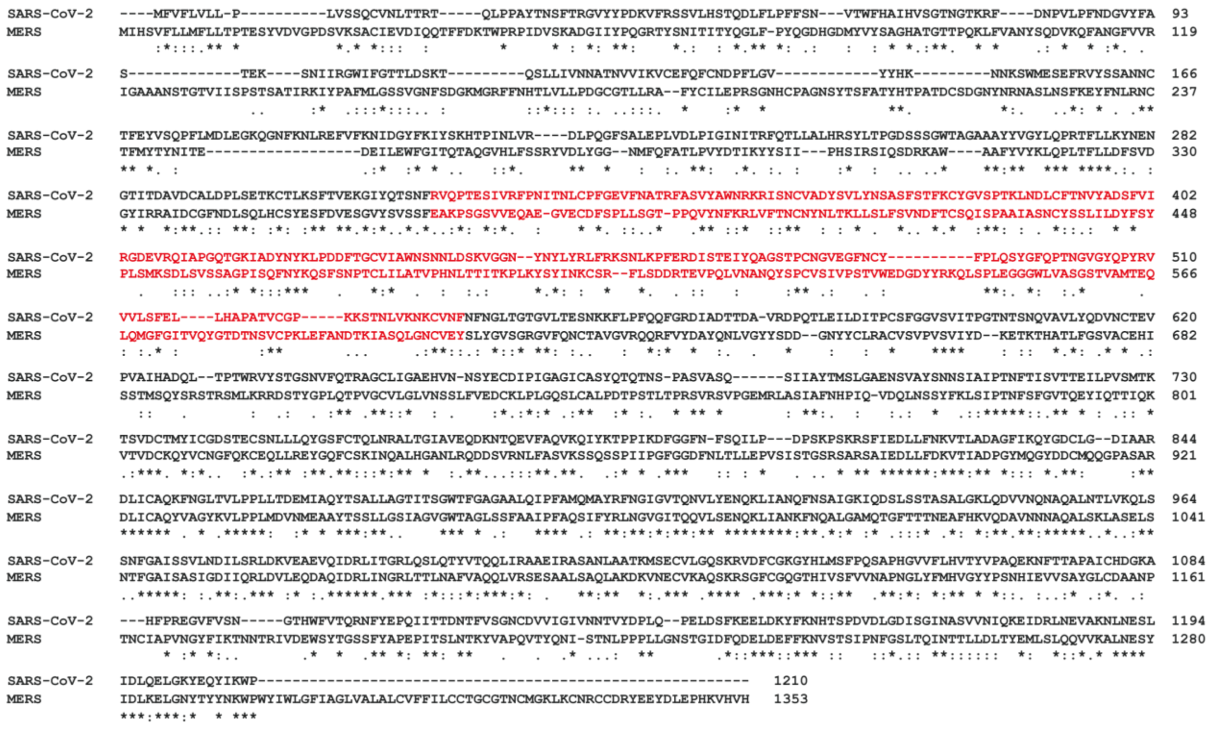

Supplementary Figure S3: Amino acid sequence alignment of spike (S) proteins from SARS-CoV-2 and MERS-CoV. RBD regions (SARS-CoV-2, residue 319-541 and MERS, residue 367-606) are highlighted in red. The figure was created with ClustalW2 and reformatted in Adobe Illustrator CC 2020.

What is your age?	20-40	123/206
	40-60	75/206
	>60	8/206
What is your gender?	Male	98/204
	Female	106/204
Do you believe you have experienced any COVID-19 symptoms in the past 6 months?	Yes	36/205
	No	169/205
Have you or any members of your household traveled outside Okinawa in the past 6 months?	Yes	64/206
	No	142/206

Supplementary Table ST1: Summary of Optional Survey Results. 206 participants among the 675 processed barcodes opted to take a short, optional survey. Participation was voluntary.

Supplementary Methods

ID generation and reporting of results

Each sample ID was stored in a database table along with values representing test results and disclaimer acceptance. The web application queried this database to retrieve the status of the requested sample ID. The sample IDs were composed of six characters generated randomly from a set of alphanumeric characters (a-zA-Z0-9) (random string generator https://www.random.org/strings/), excluding letters O, I, l, Z, Q and numbers 0, 1, 2, 9 to minimize human read errors. Out of approximately 10^{10} combinations, 1000 unique IDs were randomly selected. Some additional security features, such as request rate limiting, were implemented to restrict the use of automated tools to retrieve results for all possible sample IDs. Basic functions were developed to allow batch import and export of test results in a delimited text format, allowing easy transfer of data between the testing system and the web application database.
